# Organizational Culture and Implications for Workplace Interventions to Reduce Sitting Time Among Office-Based Workers: A Systematic Review

**DOI:** 10.3389/fpubh.2018.00263

**Published:** 2018-09-24

**Authors:** Wendell C. Taylor, Richard R. Suminski, Bhibha M. Das, Raheem J. Paxton, Derek W. Craig

**Affiliations:** ^1^Department of Health Promotion and Behavioral Sciences, Center for Health Promotion and Prevention Research, School of Public Health, The University of Texas Health Science Center at Houston, Houston, TX, United States; ^2^Department of Behavioral Health and Nutrition, Center for Innovative Health Research, University of Delaware, Newark, DE, United States; ^3^Department of Kinesiology, East Carolina University, Greenville, NC, United States; ^4^Department of Community Medicine and Population Health, University of Alabama, Tuscaloosa, AL, United States

**Keywords:** organizational culture, sit less, workplace intervention, sitting time, sedentary behavior, prolonged sitting, workplace culture, culture of health

## Abstract

**Background:** Time spent in sedentary behaviors is an independent risk factor for several chronic diseases (e.g., cardiometabolic diseases, obesity, type 2 diabetes, and hypertension). Recently, interventions to reduce sitting time at work (a prominent sedentary behavior) have been developed and tested. Organizational culture plays a critical role in the success of workplace interventions. However, there are a limited number of studies that have examined the role of organizational culture in reducing sitting time in the workplace.

**Objectives:** Therefore, in this systematic review, we summarized the empirical literature investigating organizational culture and sedentary behavior in the workplace and identify gaps in the knowledge base.

**Methods:** We described the procedures of our systematic review and included two study flow diagrams that detailed the step by step process. Combinations of several search terms were used; the databases searched were PubMed, Medline, Academic Search Complete, and Google Scholar. We started with thousands of citations. After applying the inclusion and exclusion criteria, eight relevant articles were identified.

**Results:** For each identified article, the data extracted included citation, sample, objective, intervention, assessment of organizational culture and workplace sitting, findings, and implications. Each article was rated for risk of bias by population, intervention, comparator, outcomes, and study design (PICOS) analysis. The classification for each study was either: high-, moderate-, or low-quality evidence. Given the paucity of data, no definitive conclusions were presented; however, positive trends were highlighted.

**Conclusions:** Work place interventions to reduce sitting time at work may benefit from considering elements of organizational culture; however, the evidence to date is sparse and more high-quality studies in this area are needed. To advance the field of workplace health promotion, organizational culture, and interventions to reduce sitting at work, we present 11 recommendations.

## Key concepts

**Organizational culture:** Organizational culture is the shared values, beliefs, or perceptions held by employees within an organization or organizational unit (1).
**Sedentary behavior:** Any waking behavior characterized by energy expenditure ≤ 1.5 metabolic equivalents, while in a sitting, reclining, or lying posture (2).
**Culture of health (COH):** Environments with a culture of health, place value on, and are conducive to, employee health and well-being (3). A concern for employee health must permeate all aspects of an organization and its corporate identity (4).

## Introduction

On average, Americans spend ~9 h sleeping or engaging in personal care activities (5). In addition, Americans spend ~8 h working or doing work-related activities; therefore, one-half of waking hours are related to work (5). So, what happens at work has undeniable consequences for physical, psychological, emotional, and social well-being. Unfortunately, in the workplace, sitting remains the dominant posture for office-based workers. Although there are detrimental health consequences associated with prolonged sitting, limited data exist on the variety of factors that contribute to this cultural norm.

### Rationale

High levels of sitting are associated with increased risk of several adverse health outcomes including diabetes, heart disease, type-2 diabetes, and obesity. Sedentary behavior may be an independent risk factor for chronic diseases especially among adults with insufficient or low physical activity levels (6–8).

To reduce sedentary behavior in the workplace, understanding the influence of organizational culture on these behaviors is critical. Even though there are different definitions of organizational culture, an accepted definition is artifacts, espoused beliefs and values, and underlying assumptions (9). Artifacts are behaviors, rituals, language, myths, and dress. Espoused beliefs and values are typically reported by management as core to the organization. Underlying assumptions relate to organizational life and illuminates why organizational members go about their day-to-day work lives as they do (9). In essence, organizational culture is enduring, stable, and can take a long time to develop. In simple terms, organizational culture represents shared basic assumptions, values, and beliefs that characterize a setting and are taught to newcomers as the proper way to think and feel as employees of the organization.

### Objectives

The importance of organizational culture and health outcomes has been documented. There is evidence that health promotion programs that incorporate more cultural elements in their strategies result in a reduction of employee health risks by as much as 5% per year, a level 2.5 times greater than health promotion programs without a cultural component (10). Another study found that most organizations with cultural support (66%) reported greater improvements in health behaviors compared to organizations with little or no cultural support (26%) (11). From a related perspective, organizations with very supportive leadership were almost 4 times more likely to report substantial improvements in employee health risks and 2.5 times more likely to report substantial improvements in medical cost trends. Importantly, the inverse was true. Organizations with minimally supportive leadership were ~4 times more likely to report minimal improvements in both employee health risks and medical cost trends (12). Based on existing literature, we hypothesize that organizational culture can hinder or enhance intervention programs to reduce sedentary behavior in the workplace.

### Research questions/specific aims

Therefore, to reduce sedentary behavior, the implications and effects of organizational culture on sedentary behavior interventions merit systematic and thorough investigation. We found no reviews that specifically addressed this important topic. To fill this critical gap in our knowledge base, the objectives of this paper were to: (1) identify and describe the current literature related to organizational culture and sitting behavior at work; (2) identify gaps in the knowledge base; and (3) provide recommendations to advance the field related to reducing sedentary behavior among office-based workers.

## Materials and methods

### Study design

All study designs were included and evaluated. The designs included randomized controlled trials (RCTs), cross-over RCTs, cluster-randomized controlled trials (cluster-RCTs), quasi-RCTs of interventions, non-randomized controlled trials, focus groups, and structured interviews.

### Participants, interventions, comparators

The participants were any employees at the workplace. The interventions were designed to reduce sitting at work. The comparators were employees at the workplace who did not participate in the intervention.

### Systematic review protocol

The systematic review protocol involved independent reviews of identified articles by study investigators. Then consensus was achieved as to conformity with the inclusion and exclusion criteria. There were no disagreements that required a third party to adjudicate.

### Search strategy

**Figure 1:**
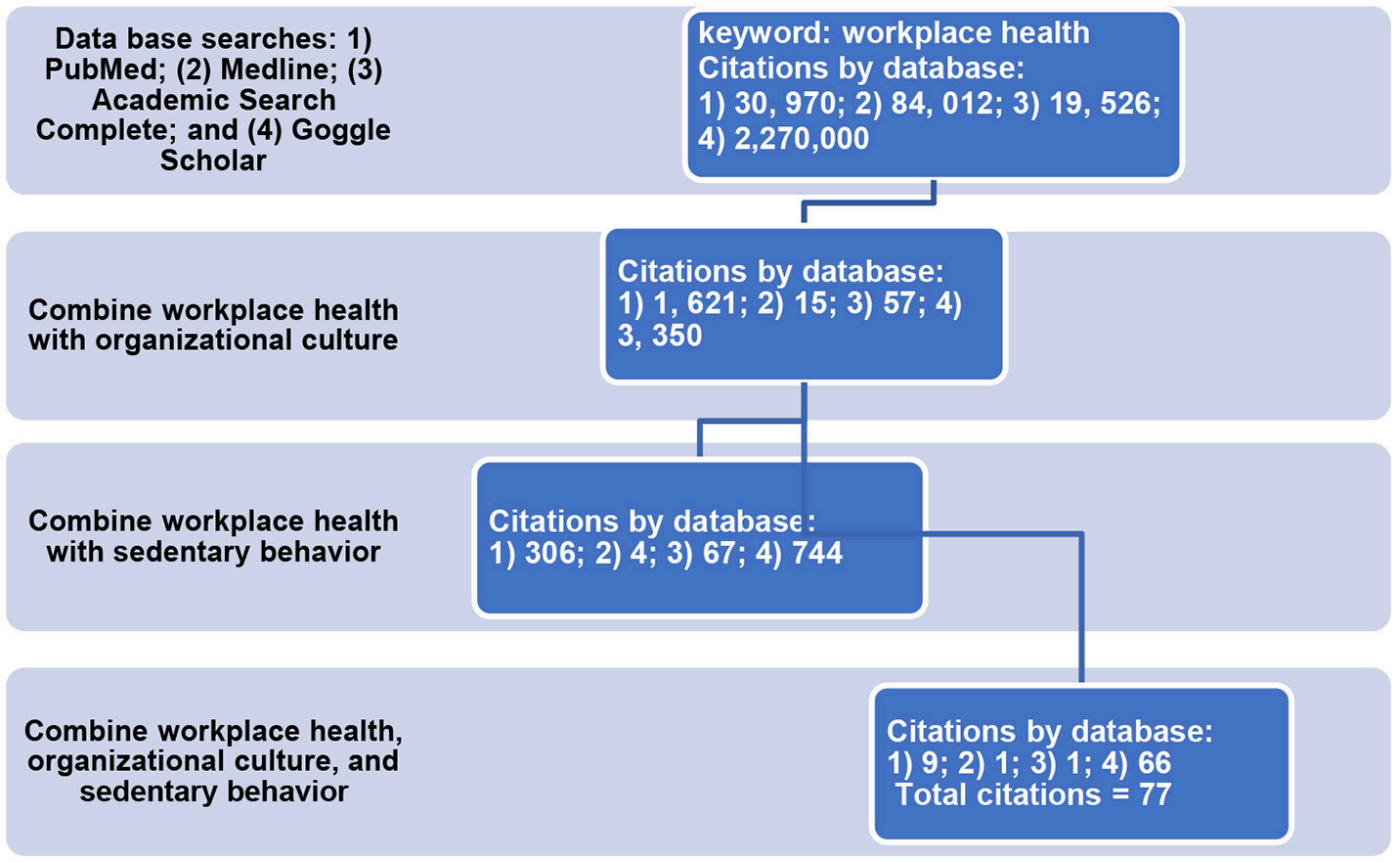
Study flow diagram one: sequence of database searches. Keywords: workplace health, organizational culture, and sedentary behavior. Inclusion criteria for systematic review: primary data collection from employees or employers; data related to organizational culture and sedentary behavior. For Medline, we used the search term workplace health promotion instead of workplace health to meet the accepted terms for the database.

**Figure 2:**
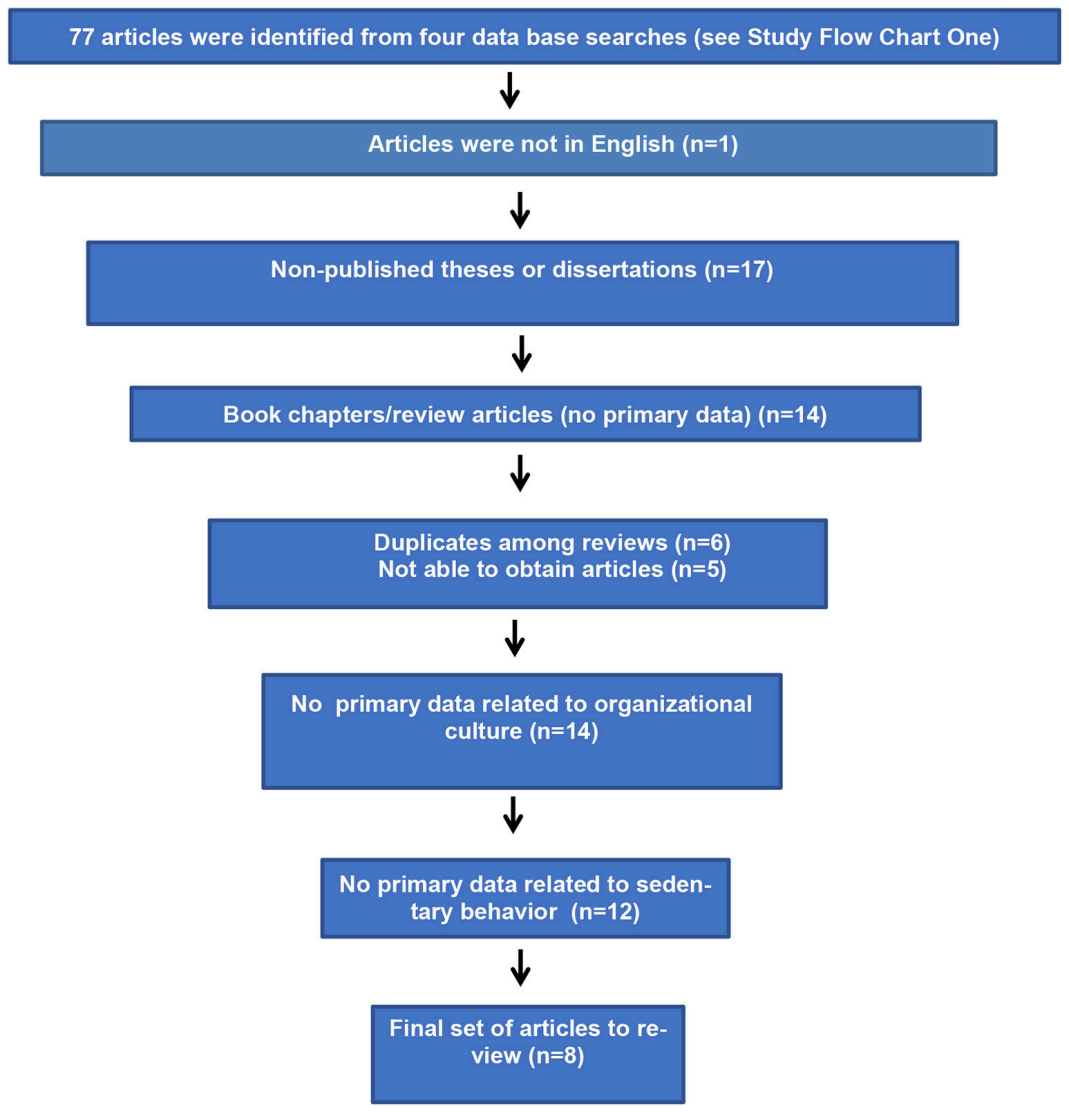
Study flow diagram two: apply inclusion and exclusion criteria to 77 articles identified from four data searches (see Flow Chart One). Inclusion criteria for systematic review: primary data from employees or employers and primary data related to organizational culture and sedentary behavior. Exclusion criteria for systematic review: non-English language, thesis or dissertation (not published in peer-reviewed journal); unable to obtain publication; no primary data related to organizational culture and sedentary behavior.

The two study flow diagrams represent the major search strategy with four databases (PubMed, Medline, Academic Search Complete, and Google Scholar) and three key search terms—workplace health, organizational culture, and sedentary behavior. The inclusion criteria were primary data collection for organizational culture and sedentary behavior in the workplace. The exclusion criteria were non-English language study; theses or dissertations (not published in peer-reviewed journals); unable to obtain publication; and no primary data related to organizational culture and sedentary behavior in the workplace. Time frame was not limited given the novelty of research in this area. In addition to the main search strategy presented in Study Flow Diagrams One and Two (Figures 1, 2), we conducted additional searches using combinations of key search terms from the following Medical Subject Headings (MeSH) terms: workplace culture, workplace, worksite, sitting, occupational sitting, and sedentary behavior. Furthermore, we reviewed the references of relevant articles such as review papers. We found no other appropriate references to add to the articles retrieved from the main search strategy.

### Data sources and data extraction

The data sources were: PubMed, Medline, Academic Search Complete, and Google Scholar. For each article, the data extracted included citation, sample, objective, intervention, assessments of organizational culture and sitting time, findings, and implications (Table 1). Each article was rated for risk of bias by population, intervention, comparator, outcomes, and study design (PICOS) analysis. The classification of each article was either: high-, moderate-, or low-quality evidence.

**Table 1:** Organizational culture and sitting at the workplace: literature review.

**References**	**Sample**	**Objective**	**Intervention**	**Assessment of organizational culture**	**Assessment of sitting**	**Findings**	**Implications**
Adeleke, et al. (13)	•Regional office of a large public sector service delivery organization in Australia. •79 desk-based employees (26% of 300 possible) •Participants were mostly female (83%) and between 30 and 59 years of age (86%). Most had completed high school (83%), worked full time (60%), and had been employed at the organization for at least 3 y (68%). •Problem: High employees work place sitting time	Evaluate the effect of installing sit–stand workstations on their employees' workplace sitting time.	•Installation of electronic and fully adjustable sit–stand desks received information on how to use and adjust the workstation. •The intervention was conducted at a workplace that had previously implemented an intervention to reduce sedentary time at work. •The previous intervention involved educational materials aimed at encouraging workers to stand more at work.	Self-report survey on commitment of the workplace to their health and choice to stand and move at work	Self-report of work place sitting time using the Occupational Sitting and Physical Activity Questionnaire	No comparison group or alternative to the intervention 3-months post-baseline: - Sitting time decreased from 17% of work hours spent sitting to 13% (-80 min/8 h workday). - Standing time at work increased from 12% – 19% (+72 min/8 h workday). No significant effect on sitting time during nonworking hours	Participants agreed or strongly agreed that the workplace was: •Supportive of their health (84%) •Supportive of staff choice for standing and moving at work (74%). •The intervention was conceived and implemented entirely by workplace staff.
Brakenridge et al. (14)	Desk-based office workers from one organization randomized to: 1) Organizational support only (n = 46/87 completed intervention) 2) Organizational support + activity tracker (*n* = 25/66 completed intervention)	Evaluate the impact of organizational support strategies alone or with activity monitoring on sitting time at work.	Stand Up Lendlease, 12-month intervention to reduce sitting time at work.	•Survey used to assess the following work-related outcomes: •Job performance •Job control •Work satisfaction	ActivPAL3 monitors used to ascertain sitting time during work- and overall hours, prolonged sitting time (≥30 min bouts), time between sitting bouts, standing time, stepping time, and number of steps.	•Baseline (mean ± SD) 74.3 ± 9.7% of workday sitting, 17.5 ± 8. % standing and 8.1 ± 2.7% stepping. •No significant change in 3-month outcomes. •Significant (*p* < 0.05) reductions in both groups at 12 months in sitting time at work (−37 min/10 h), prolonged sitting at work (−43 min/10 h) and standing at work (+33 min/10 h) •After adjusted for confounders, the only significant between-group differences were a greater stepping time and step count for Group ORG + Tracker relative to Group ORG (+20.6 min/16 h day; +846.5steps/16 h day) at 12 months.	•Intervention using organizational support strategies to reduce sitting time at work are effective when they are worksite-driven and internally delivered. Minimally intensive nature of intervention is a positive.
DeJoy et al. (15)	The present analyses were based on 1859 employees of Dow Chemical Company.	To evaluate the comparative effectiveness of environmental weight loss intervention alone vs. in combination with an individual intervention	The moderate intensity condition utilized a set of inexpensive and widely applicable environmental modifications aimed primarily at creating a more supportive work environment for physical activity and healthy eating. The high-intensity condition included all of the moderate interventions and several additional elements designed to engender a relatively high level of management engagement and support for the weight management goals of the project.	Environmental Assessment Tool was used to check the effectiveness of the environmental manipulation (moderate vs. intense to influence organizational culture).	Assessment of physical activity/inactivity based on a standardized Health Risk Assessment instrument. [Conceptual and measurement distinctions between physical inactivity (outcome in article) and sedentary behavior (inclusion criterion for this review) have been reported (16). Nonetheless, we chose to include this article because of its emphasis on changing organizational culture with moderate and intense environmental interventions].	Employees who participated in the individual program were no more successful at losing weight than those exposed to only the environmental interventions. Those participants in the individual program at the environmental intense sites were 1.87 times more likely than non-participants to reduce their risk of physical inactivity (*P* = 0.0082).	•Based on process data, the authors concluded that management support must be effectively communicated at levels of the organization most notably to rank-and-file employees.
Hadgraft et al. (17)	Convenience sample of 10 men and 10 women. Employees and managers in retail, health and IT industries with no formalized programs to reduce sitting.	Explore barriers to reducing office workplace sitting, and the feasibility and acceptability of strategies targeting prolonged sitting in the workplace	None	Semi-structured interviews covering barriers to reducing sitting, the feasibility of potential strategies aimed at reducing sitting, and perceived effects on productivity	•The Occupational Sitting and Physical Activity Questionnaire •Outcome: Percentage of time at work spent standing, sitting, and moving during a typical work day in the past week.	•Median 7.2 h/d spent sitting at work •Sitting time at work related to: •Reliance on computers for work •Workload •Having furniture designed for a seated posture •Standing, stretching and/or moving about the office related to: •Concerns about appearance (e.g., looking “weird”) •Needs to reduce sitting time at works were: •Low cost strategies (e.g., standing meetings) •In-person communication about reducing sitting time	•Low-cost strategies (e.g., standing) may be feasible and acceptable approaches to reducing sitting time at work. •Barriers to uptake that should be considered are social norms and workload •Two key elements that seem essential for work-related sedentary behavior change to occur are: -Raising worker awareness about the health risks of sitting at work
						•Evidence supporting a business case to reduce sitting at work (noted by managers).	•-Building supportive organizational cultures •The business case for reducing sitting at work needs to be strengthened through research on productivity and cost-effectiveness
Hadgraft et al. (18)	•Office-based workers at 14 government worksites •121 intervention and 87 controls completed 3-month follow-up •97 intervention and 70 controls completed 12-month follow-up •Mean age 45.6 ± 9.4 y 68.4% female, 79.4% Caucasian, 66.8% had post-school education, and 79.2% worked full-time	•Assess the impact of the intervention on four social-cognitive constructs •Examine if the four constructs mediated intervention effects on workplace sitting	•Stand Up Victoria •Organizational, environmental and individual level approach to reduce workplace sitting time.	Self-administered online questionnaire measuring: Perceived organizational norms Perceived organizational/social support for sitting less at work.	ActivPAL3 activity monitor used to measure workplace sitting time	•No significant effect on perceived organizational norms at 12 months •No relationship between changes in perceived organizational norms and changes in workplace sitting time at 3 and12 months •Self-efficacy for overcoming barriers to sitting less at work mediated intervention effects on workplace sitting time (indirect effect: −10.3 min/8 h day; 13.9% of total effect)	•Interpersonal and environmental levels of influence on initiating and maintaining workplace sedentary behavior change need to be better understood to improve intervention development and refinement.
Healy et al. (19)	43 participants	Changes in minutes/day at the workplace spent sitting (primary outcome)	4 week intervention with organizational, environmental, and individual components	Assessed organizational support for successful intervention adoption with consultation with company representatives and management.	ActivPAL3	•Compared to controls, intervention group significantly reduced workplace sitting	•Organizational change evidenced through workplace social norms and workplace culture is likely to take longer than a 4-week study to become institutionalized.
Flint et al.(20)	Convenience sample of 21 office workers from small to medium-sized UK companies	Explore employees' perceptions of sitting time	None	Four focus groups comprised of non-managerial employees and one focus group of managers. Asked about: Perceived association between sitting time and health. Strategies that could be used to break up or reduce prolonged sitting at work.	Workforce Sitting Questionnaire International Physical Activity Questionnaire (IPAQ) short form	•Mean sitting time was 6.4 h/d •Employees indicated they: •Sat too long at work •Know about the link between prolonged sitting at work and increased risk of chronic health problems •Barriers to Sitting Less and Moving More •Sitting was integral to their occupational role •Organizational culture •Lack of motivation •Physical environment (e.g., lack of sit-stand desks) •Poor quality recreational facilities on-site and no managerial support for using it •Strategies to Reduce Sitting at work •Organizational support and encouragement to reduce sitting time at work •Have employer provide evidence-based information on the risks of sedentary behavior •Incentives •Allowance to take breaks at work	•Organizational culture should be considered in the design and implementation of workplace interventions to reduce sitting time. •Personal determinants and the workplace environment also play key roles in reducing employees' sitting behavior at work.
Such and Mutrie (21)	•Seven men and six women who had participated in the Sit Less and Walk More intervention. •All were engaged in predominantly desk-based work.	Examine the organizational cultural factors that impede and promote reduced sitting time in the workplace.	•“Sit Less” health promotion intervention with the following components: •Sit Less and Walk More' 4-week, pedometer intervention •Awareness-raising sessions highlighting issue of sitting time at work. •Posters encouraging physical activity placed at key decision-making points around the workplace	•Face-to-face in-depth interviews •Analyses of four workplace policy documents •Employee workplace handbook •Employee mental health and wellbeing statement •Health and safety components of the organization's annual reports •Organization's strategic plan and performance framework.	•A ‘sit less and walk more' 4 week intervention. Thirty-five volunteers took part •In this phase: They were provided with pedometers to record steps taken at work. •Baseline measures were taken in week one. This was followed by a one-to-one counseling intervention which aimed to help participants identify where, how and when sitting time could be reduced and step counts increased. Steps were then recorded for a further 3 week period.	•More sitting at work related to: •Greater work demands •Policy, rules and regulations such as working from home and home flexi-time (both *encouraged* more sitting) •Working in silos limited chance to move around work space •Issues such as family life, transport, and the nature of the modern working environment •Lack of formal practices and mention of sitting time in organization's documents.	•To effectively reduce sitting time at work it is important to consider a range of structural, organizational and cultural factors and their dynamic interactions

### Data analysis

From the main search strategy, two study flow diagrams show that we began with thousands of citations, applied all three keywords, and identified 77 potentially relevant articles. The inclusion and exclusion criteria were applied to the 77 articles and 8 relevant publications were identified to include in the data analysis. We reviewed eight articles and the data extracted were: citation, sample, objective, intervention, assessments of organizational culture and sitting time, findings, and implications (Table 1). Then, each article was rated based on high-, moderate-, or low-quality evidence and described in the text of the results section.

## Results

### Provide a flow diagram of the studies retrieved for the review

Study flow charts one and two represent the main search strategy from beginning to end. We started with thousands of citations and applied the inclusion and exclusion criteria which resulted in 77 articles; the final set included 8 articles.

### Study selection and characteristics

The eight articles were summarized and evaluated based on population, intervention, comparator, outcomes, and study design (PICOS). High-quality evidence is characterized by studies with large sample sizes that are racially and ethnically diverse. An appropriate comparison group is identified. The study designs are RCTs, cross-over RCTs, cluster-randomized controlled trials (cluster-RCTs), and quasi-RCTs of interventions. The studies with high-quality evidence have low risk of bias. Low-quality evidence is characterized by studies with small sample sizes, no comparison group, demographically homogeneous participants, no or flawed intervention, and weak study design (e.g., pre- and post-single sample). The studies with low-quality evidence have high risk of bias. One purpose of focus groups is to generate hypotheses for future studies. Nonetheless, from the perspective of the PICOS framework, focus group studies are low-quality evidence. Moderate-quality evidence is characterized by studies that do not meet the criteria for high quality evidence but are promising and superior to studies with low quality (e.g., non-randomized controlled trials). The studies with moderate-quality evidence have medium risk of bias.

### High quality evidence

In a cluster-randomized controlled trial with 153 desk-based workers, organizational-support strategies designed to reduce sitting in office workers with and without an activity tracker were evaluated (14). The authors assessed the short-term (3 months) and long-term (12 months) effectiveness of the intervention. The organizational-support strategies consisted of a wellness champion choosing from a menu of choices and selecting options appropriate for each organization. The options included: an information booklet about sitting and health implications; five nightly emails with activity-promoting tips and images of active participants; participation of senior executives was communicated to employees; the workplace champion presented at least 10 workplace presentations, and informal discussions with managers continued throughout the study. The average time spent sitting during work and overall were the primary outcomes. A secondary outcome was the number of steps per day.

With or without an activity tracker, organizational-support strategies showed improvements in sitting and standing. At 3 months, both interventions resulted in small and non-significant differences in sitting time and activity outcomes. At 12 months, both interventions resulted in statistically significant reductions in sitting time. The activity tracker condition showed increases in overall stepping time and step counts. Improvements were most evident at 12 months. The authors concluded that embedding changes in an organization is not a rapid process and it takes time to generate organizational culture change. Furthermore, behavioral improvements can occur without environmental modifications. This study was the first to report the impact of organizational support and activity tracker strategies on office workers' sitting time (14).

In another study, building on a multi-component trial that successfully reduced sitting in the workplace, the purpose was to provide insights into the mechanisms (mediators) to explain the results (i.e., reduced sitting) by examining short- and long-term mediation effects (18). Two hundred and thirty-one office-based workers were randomly assigned to intervention and control conditions by worksite. Workplace sitting was measured with the activPAL3 device. Participants reported spending 7.2 h (median) of their working hours sitting. The intervention was composed of organizational, environmental, and individual level components. The organizational level intervention was tailored management emails. To evaluate perceived organizational norms, levels of agreement were assessed to the statement; “My workplace is committed to supporting staff choices to stand or move more at work.” Based on mediation analysis, social norms around appropriate workplace behavior and workload pressures were perceived as barriers. Furthermore, the intervention significantly improved perceived organizational norms at 3 months but not at 6 months. The authors noted that the organizational level intervention designed to improve workplace culture was discontinued at 3 months. It was recommended that future workplace interventions invest in longer organizational cultural change strategies to sustain perceived cultural changes related to moving more and sitting less (18).

A quasi-experimental research design, with 1,859 employees, was used to compare the outcomes for two levels of environmental interventions and for participants who did or did not simultaneously self-select into an individually focused weight loss intervention (15). In essence, the comparative effectiveness of environmental weight loss interventions alone vs. in combination with an individual intervention was evaluated. The outcomes of interest were weight loss and physical activity/inactivity. The goal for the moderate environmental interventions was to utilize a set of simple, low cost interventions that would be easy to implement in a wide variety of workplaces and that could be sustainable over time. The intense environmental condition was designed to engage leadership and create a more positive and supportive climate for health. The individually focused program consisted of setting a weight-related goal and reporting baseline weight at sign up and providing self-reported weight information three times after signup at 3-month intervals. The results were that employees who participated in the individual intervention were no more successful at losing weight than those exposed to only the environmental interventions. However, those who participated in the individual program at the intense environmental intervention sites were 1.87 times more likely than non-participants to reduce their risk of physical inactivity (*P* = 0.0082).

The moderate and intense environmental conditions differed significantly across time in terms of supports for nutrition and weight management and general organizational supports; the environmental manipulation was effective with some fading during year 2 of the intervention. However, the authors noted that leadership engagement failed to impact frontline employees at a subjective or perceptual level. The recommendation from this study was that management support must be effectively communicated so that visible actions and tangible changes that impact the daily work life of rank-and-file employees can be observed. Environmental interventions may be most effective when they are publicized through multiple channels and accompanied by policy actions that reinforce the desired behaviors. [An explanatory note about the inclusion of this article in our review; conceptual and measurement distinctions between physical inactivity (outcome in article) and sedentary behavior (inclusion criterion for this review) have been reported (16). Nonetheless, we included this article because of its emphasis on changing organizational culture with moderate and intense environmental interventions].

### Moderate-quality evidence

During a 4-week, two arm, non-randomized controlled trial, an intervention with organizational, environmental, and individual elements was implemented (19). The primary outcome of interest was workplace sitting time. There were 43 participants. The organizational support included emails providing “standing tip of the week” and a workshop focusing on the health consequences of excessive sitting. Organizational support strategies were adjusted based on consultation with organization's representatives and management. Environmental strategies were the installation of dual display sit-stand workstations. The individual interventions were an initial 30-min face-to-face consultation between a health coach and each intervention participant followed by three telephone calls (one per week). The sessions emphasized behavior change strategies such as goal setting, self-monitoring, and problem solving. In comparison to the control group, the intervention group significantly reduced workplace sitting time; workplace sitting was almost exclusively replaced by standing. Because the intervention period was 4 weeks, the authors reported that most of the change was attributable to environmental and individual strategies; however, it was acknowledged that organizational support was essential. The authors concluded that organizational strategies to change workplace social norms and workplace culture will take longer that a 4-week intervention to become institutionalized (19).

### Low quality evidence (i.e., convenience sample, focus group, and interview studies)

A study examined the impact of installing sit–stand workstations on their employees' sitting time at work (13). The intervention was conducted with a convenience sample of 79 desk-based employees working at a large, service delivery organization in Australia. Employee perceptions of organizational culture and sitting time at work were assessed before and after the employees were given sit-stand work stations. No comparison group was used. Results were positive and indicated that the intervention decreased sitting time at work by an average of 80 min/8 h workday and increased standing time by an average of 72 min/8 h workday. In addition, employees believed that the workplace was supportive of their health (i.e., organizational culture) and the employees had a choice of standing and moving at work. This study was deemed low-quality evidence because it had many limitations related to its study design (e.g., small percentage of staff participated) and no comparison group.

In another research project, the volunteers for a qualitative sub-study were part of a larger study that included pedometers to record steps and one-to-one counseling sessions to reduce sitting and increase step counts (21). Based on a model of organizational culture, qualitative, face-to-face, in-depth interviews were conducted with 13 volunteers. The model of organizational culture included the constructs of values and belief system, strategy, structure, and operations affected by the external environment. The findings from the qualitative study were that the “inevitability of time pressure” and the work ethic to “get things done” undermined the motivation to reduce sedentary behavior. It was recommended that the organization appoint a “champion for sitting less.” Basically, the norm of sedentary time was unchallenged by the work hierarchy (21). The authors concluded that to change sitting as a norm, a whole systems approach is needed that includes workplace policy and norm-changing interventions. Co-produced interventions (employees, employers, management and scientists working in partnership) can challenge sedentary behavior at different and interconnected organizational levels.

In another qualitative study, with a convenience sample of 21 employees, self-reported occupational sitting time was 6.4 h (20). The purpose of the five focus groups was to explore perceptions of health risks associated with prolonged sitting and potential strategies to reduce sitting at work. Four focus groups consisted of non-managerial employees and one focus group was exclusively managers. The majority of participants had experienced negative symptoms associated with sitting at work including neck and back pain, poor posture, weight gain, and reduced concentration. In addition to personal determinants, employees highlighted the key role of workplace environment and organizational culture in reducing sitting behavior at the workplace. One of the barriers to reducing sitting time was the chargeable time culture; everything needs to contribute to the organization's productivity. One respondent cited “Sit at your desk for a lunch break and eat while working.” To change culture, it was recommended to have corporate endorsement of well-being champions and communication at all levels within the organization (20).

In a different qualitative study (semi-structured interviews), with a convenience sample of 20 participants from three organizations, the objective was to assess perceptions about reducing workplace sitting (17). None of the workplaces had implemented any formal interventions to reduce prolonged sitting time. The purpose of the study was to identify barriers to reduce sitting and perceptions about a range of sitting reduction strategies. On average, participants reported sitting at work 7.2 h per day (minimum was 4.0 h and maximum was 9.5 h). The three prominent barriers to reduce workplace sitting were: the nature of work, organizational social norms, and office furniture and layout. Organizational social norms were perceptions of what was considered normal as workplace behavior. Related to organizational influences, the respondents were asked: “What level of priority do you think your organization places on reducing sitting time at work.” The authors concluded that building a supportive organizational culture and raising awareness of the adverse health effects of prolonged sitting may be important to improve individual level and other strategies for change (17).

### Synthesized findings

In summary, the empirical database is limited; no definitive conclusions are presented. The majority of the studies were focus groups and qualitative interviews with data to generate hypotheses for interventions. Basically, more intervention studies are needed. Our preliminary assessments to be confirmed with additional studies are that workplace interventions to reduce sitting time: (a) can be low-cost (e.g., stand rather than sit at meetings); (b) should include efforts to raise awareness regarding the negative health effects of prolonged sitting at work; (c) need to target multiple levels (e.g., employees, employers, individual, social, environmental, and organizational); (d) need to have strong and clear components that focus on organizational culture and; (e) should make a business case (e.g., increased productivity) related to reduced sitting at work.

### Risk of bias

In a recent Cochrane review (22), risk of bias was assessed in the following domains: random sequence generation, allocation concealment, blinding of participants and personnel, blinding of outcome assessment, incomplete outcome data, selective outcome data, validity of outcome measure, and baseline comparability/imbalance for age, gender, and occupation of groups. Each potential source of bias was graded as high, low, or unclear with justification for the judgments. These eight domains have marginal relevance and applicability to a database with eight studies. There were three high-quality evidence papers; one moderate-quality evidence paper; and four low-quality evidence papers. The low-quality evidence papers were primarily focus group studies or semi-structured interviews. Given the limited database and predominance of qualitative studies, the risk of bias is unclear.

## Discussion

### Summary of main findings

As noted in the Introduction section, definitions of organizational culture are essential to workplace health promotion. A clear definition is needed for the field to move forward. A pioneer in the study of organizational culture, Schein (23) in his recent book, presents a dynamic definition of organizational culture.

“The culture of a group can be defined as the accumulated shared learning of that group as it solves its problems of external adaptation and internal integration; which has worked well-enough to be considered valid and, therefore, to be taught to new members as the correct way to perceive, think, feel, and behave in relation to those problems. This accumulated learning is a pattern or system of beliefs, values, and behavioral norms that come to be taken for granted as basic assumptions and eventually drop out of awareness” (page 6) (23).

What is noteworthy about this definition from the perspective of researchers and practitioners is that there are distinct levels of observability for learned patterns of beliefs, values, assumptions, and behavioral norms. Of the constructs noted above, behavioral norms are the most visible and represent an expression and manifestation of assumptions, values, and beliefs.

Given this definition, the primary conclusion from this review is that literature documenting the role of organizational culture on sedentary behaviors at work is sparse (i.e., only eight studies) and fairly recent (i.e., six of eight papers published since 2016). The outcomes, however, from the available studies suggest that efforts to reduce sitting time at work are promising when organizational culture is considered. Further research in this area is warranted. Therefore, we present 11 recommendations to guide and advance the field.

### Recommendations

- Mechanisms, mediators, and processes that influence organizational culture include leadership, organization's mission statement, management and strategic communication, management behavior, employee autonomy/empowerment, coworker support, employee engagement, values, belief systems, environmental supports, policies, and practices (24). Similar to an earlier study (24), we recommend identifying what matters most for changing how employees think and feel about the organization's support for health and reducing sedentary behavior. The potential targets are policies, environmental supports, and clear and favorable upper- and mid-management communication.
- In a comprehensive review of the measurement of workplace culture, most instruments did not focus on workplace *health* culture (12). Instead, subscales related to the culture of health were identified. One exception is the Lifegain Wellness Culture Survey (also known as the Lifegain Health Culture Audit), which measures five core dimensions of culture—values, norms, touch points, peer support, and climate (11). The validity and reliability of this instrument have been measured in several studies (11). Instruments related to the overall culture of the organization include: The Organizational Culture Inventory, the Denison Organizational Culture Survey, and the Organizational Culture Profile (25). The challenge of measuring organizational culture has been noted by several authors (25). Most quantitative measures of culture capture the espoused values and/or behavioral norms in organizations and not the full richness of the construct—including myths and stories. A focus on intangibles related to organizational culture makes a complete reliance on quantitative approaches unsatisfactory, emphasis should be placed on qualitative assessments (25).
- Organizational culture should be studied in conjunction with organizational climate. Climate and culture can be mutually supportive. Climate is more sensitive to workgroup norms and highly variable across an organization, whereas culture is more enduring and stable across the entire organization (12). Organizational climate is defined as the mood or unique “personality” of a workplace (26). Organizational climate, also, has been defined as the shared perceptions of and the meaning attached to the policies, practices, and procedures employees experience and the behaviors they observe getting rewarded and that are supported and expected (25). On the other hand, as noted earlier, organizational culture is defined as the shared basic assumptions, values, and beliefs that characterize a setting and are taught to newcomers as the proper way to think and feel and are communicated by the myths and stories people tell about how the organization came to be the way it is (25). In this paper, we focused on organizational culture because it is more enduring and stable than organizational climate. For example, a charismatic leader affects the organizational climate. When a charismatic leader departs a company the organizational climate can change dramatically; however, the organizational culture remains basically intact. Nonetheless, the relationships between climate and culture can be mutually reinforcing and both merit empirical analysis.
- Organizational culture should be studied as potential antecedents, consequences, moderators, and mediators for interventions designed to reduce sedentary behavior. In previous research, organizational culture was studied as a contextual variable that moderated relationships between and among other constructs (25). For example, can dimensions of organizational culture weaken or strengthen the relationship between organizational justice and leader-member exchanges (25)?
- In understanding culture, the socialization experiences of newcomers can be critical because typically, newcomers see everything with fewer preconceptions and fresh perspectives. If a newcomer affiliates him/herself with individuals who embody the positive values of the organizations, s/he will embrace the culture quickly. In the context of sedentary behavior, regular displays of reducing sedentary behavior and an active workplace culture will facilitate positive behavioral changes. However, the reverse is also true. Zappos is a prime and clear example of hiring according to cultural standards and insuring that newcomers embrace the culture. This company, which specializes in shoes (online sales), hires employees based on their potential to fit into the organizational culture (referred to as cultural fit interviews). Consistent with this mission, new hires are offered $2,000 to quit after 1 week if they perceive the job or company culture is not for them. The bottom line is that Zappos values its culture and does not want anyone who is unhappy or dissatisfied to disturb and disrupt the organizational culture. The objective is to have the entire corporation and brand supported by a loyal and dedicated workforce who provides great customer service (https://www.entrepreneur.com/article/249174).
- More research studying the effectiveness of integrating and combining individual approaches with cultural components to reduce sedentary behavior is needed. Thus, should organizations focus both on top-down and bottom-up approaches to create the needed paradigm shift to move behavior from one norm to another? Policy and environmental supports combined with individual level interventions need further research to understand what shifts organizational culture.
- Related to recommendation #6, what elements of an intervention are needed to embed the processes and mechanisms to change an organizational culture to a “culture of health?” Well-designed investigations are needed to determine what proportion of employee health improvement is due to cultural support (12). If shown to be reliable and valid, an assessment can be used by organizations to document improvements in the organization's culture of health. This information is valuable because it can document whether policy and environmental changes are sufficient to influence organizational culture in a meaningful way. In addition, such a tool could assist organizations in evaluating the impact of organizational culture on employee health and outcomes (12). More research in this area can be impactful.
- To what extent is workplace culture influenced by societal, cultural, or national norms? An international group of experts convened to provide guidance for employers to promote the avoidance of prolonged periods of sedentary work (27). The recommendations are: for those occupations which are predominantly desk-based, workers should aim to initially progress toward accumulating 2 h per day of standing and light activity (light walking) during working hours, eventually progressing to a total accumulation of 4 h per day (prorated to part-time hours). To achieve these recommendations, seated-based work should be regularly broken up with standing-based work, the use of sit–stand desks, or taking short active standing breaks (27). To what extent these recent guidelines are known and embraced by organizations including managers and employees has not been documented. Nonetheless, societal norms can influence a workplace's organizational culture.
- To what extent is a “culture of health” influenced by life cycles of organizations? Are newer or more established (mature) organizations more likely to have a “culture of health” or a “wellness culture?” To what extent is a “culture of health” influenced by the profitability and/or organizational effectiveness of the company? The culture of health may depend on the bottom line. If a company is struggling or still fighting for survival, then health may not be a priority. If an organization is thriving, perhaps it has greater flexibility and can embrace healthy practice policies and a culture of health. More research is needed in this area.
- The prevalence of workplaces with “cultures of health” is unknown. Large organizations with established health promotion programs may be more likely to have some level of cultural support for health. Perhaps, in mid-size and small companies, the presence of workplace wellness efforts may be less common and information regarding the existence of supportive cultures more difficult to assess. On the other hand, smaller companies may be able to create and retain a culture of health more easily than large companies that have many more employees and greater organizational complexity. The size of an organization and workplace “culture of health” merits further study (12).
- Related to reducing sedentary behavior is promoting physical activity during the workday. There have been several reviews and empirical studies related to promoting physical activity during the work day (28–38). For example, research has documented that mid-management support is critical in determining whether employees can consistently participate in 15-min, group physical activity breaks during the work day (39, 40). The lessons learned from physical activity promotion during the workday can inform research on reducing sedentary behavior during the workday and organizational culture change.

### Limitations

In this systematic review, an inclusion criterion was peer-reviewed published articles. We did not search sources of gray literature and unpublished studies and data; unpublished theses and dissertations were excluded. A limitation of this review is publication bias. In addition, we excluded articles published in languages other than English. Our review may be subject to language biases. Furthermore, studies with interventions that incorporated individual, environmental, and organizational elements were excluded unless specific measures of organizational or workplace culture were assessed. Some consideration of organizational or workplace culture had to be acknowledged. We found no similar reviews to compare our findings.

## Conclusions

Prolonged sitting, aggregated from work and in leisure time, may significantly and independently increase the risk of cardiometabolic diseases and premature mortality (6–8). To reduce sedentary behavior at work, organizational culture merits careful analysis. Allen presented a metaphor that workplace or organizational culture creates fertile ground in which healthy lifestyles (the good seeds), can take root and flourish (11). The organizational culture can be strong or weak. Nevertheless, organizational culture influences the meaning of workplace behaviors as well as the adoption, implementation, and effectiveness of health promotion initiatives. In a national meeting of thirty-five of the most experienced practitioners and researchers in workplace health promotion (i.e., professional think tank), four research priorities were identified. Of the four top ranked research priorities, healthy organizational culture was ranked number one as a research priority (41). The experts in health promotion and wellness concluded that a health-oriented or wellness workplace culture permits and enables healthy lifestyle choices and significantly enhances long-term program success. In other words, a strong cultural foundation sustains health-promotion initiatives in contrast to a primary reliance on targeted individual intervention strategies.

For example, prior to the development of the intervention, a formative research study was conducted to reduce workplace sitting time using height-adjustable workstations (not focused on organizational culture; thus, ineligible for our literature review) (42). A systematic assessment was implemented to inform the development of the intervention. The guiding theoretical frameworks were: Theoretical Domains Framework, Community Readiness Model, and the Behavior Change Wheel (incorporating capability, opportunity, and motivation). The assessment included eight steps. The findings were that motivation to change behavior was low because of the dominant work culture of sitting. The authors acknowledged that their comprehensive and participatory approach to identify barriers and facilitators to reduce sitting time and their focus on individual behavior change that excluded organizational policies and practices were a major limitation of their approach (42).

Substantial and enduring health promotion improvements depend upon individual initiatives and organizational culture working in partnership (i.e., mutually supportive) (11). To create a favorable organizational culture, senior leadership commitment, front-line managers' engagement, “boots-on-the-ground” personnel mobilizing grassroots efforts, strong supportive policies, health-promoting environments, and highlighting employee health as an important business objective are recommended as essential elements to “building a culture of health” in a sustained way at the workplace.

In summary, a purpose of this systematic review was to add clarity and depth to the existing literature. Even though the findings are promising from a limited database, basic questions remain: What is the evidence that strategies to reduce workplace sitting (e.g., workplace champion and management communication) influence organizational culture? What is the time frame to observe changes in organizational culture? What is the evidence that changes in organizational culture from interventions contribute to behavior change in the workplace? As more rigorous research is conducted and evaluated to address these challenges, we will move closer to achieving our goal of reducing sedentary behavior at the workplace and improving the health of all employees.

## Author contributions

WT conceived of the study. RS, BD, RP, and DC contributed to the development and design of the study. WT and RS organized the literature review. WT and RS conducted the literature review and wrote the results section. WT wrote the first complete draft of the manuscript. RS, BD, RP, and DC revised sections of the manuscript. All authors contributed to manuscript revisions, read, and approved the submitted version.

### Conflict of interest statement

The authors declare that the research was conducted in the absence of any commercial or financial relationships that could be construed as a potential conflict of interest.
